# Children’s and adolescents’ experiences of healthcare professionals: scoping review protocol

**DOI:** 10.1186/s13643-020-01298-6

**Published:** 2020-03-07

**Authors:** Gail Davison, Martina Ann Kelly, Andrew Thompson, Tim Dornan

**Affiliations:** 1 Centre for Medical Education, Queen’s University Belfast, Mulhouse Building, Mulhouse Road, Belfast, BT12 6DP Northern Ireland; 2 Department of Family Medicine, University of Calgary, 2500 University Dr. NW, Calgary, AB T2N 1N4 Canada; 3 General Paediatrics Department, Royal Belfast Hospital for Sick Children, Belfast Health and Social Care Trust, 180 Falls Road, Belfast, BT12 6BE Northern Ireland; 4 Centre for Medical Education, Queen’s University Belfast, Whitla Medical Building, 97 Lisburn Road, Belfast, BT9 7BL Northern Ireland

**Keywords:** Children, Adolescent, Experience, Paediatrics, Qualitative health research, Scoping review, Phenomenology, Child health, Health professional

## Abstract

**Background:**

Children and adolescents form a distinct patient group, whose experiences are relatively under-represented in research. Surveys have shown that healthcare professionals (HCPs) do not always communicate with them well, leaving children and adolescents under-involved and unsure who to ask when concerned. Recent qualitative studies have recognised that HCPs have a major influence on children’s and adolescents’ experiences, where poorer interactions can lead to fear, missed appointments and potentially a worse clinical outcome.

Little is known about how children and adolescents experience the HCPs who play such an integral role in their healthcare. This review aims to explore children’s and adolescents’ *lived* experience of HCPs, so that a deeper understanding of the interactions between them can equip HCPs to provide care that better aligns with patients’ needs.

**Method:**

This study will use scoping review methodology to map the existing published literature comprehensively and systematically, following a six-step framework. It will extract children’s and adolescents’ experiences, in the form of direct quotations, and thematically analyse them. The consultation exercise with children and adolescents will gather additional insights. Findings will consist of descriptions of each theme along with exemplar quotations and consultation comments.

**Discussion:**

This scoping review is unique, as it will present children’s and adolescents’ *lived* experiences of HCPs, from synthesis of their direct quotations. Findings will assist HCPs to tailor their interpersonal skills to meet patients’ needs so that better healthcare can be provided. This study will have implications for clinical educators, policy makers and guideline developers and provide suggestions for further research.

**Systematic review registration:**

Not registered

## Background

When Kelvin (age 12) winced, a healthcare professional responded

Don’t try to tell me that it hurts if I touch this, because I’ve had the same thing and I know it doesn’t hurt [1]*.*

Harvey and Jean Picker founded the international Picker Institute having found, from personal experience of terminal illness, that even scientifically, excellent healthcare could fail because it was unresponsive to patients’ individual needs [2]. The guiding principle of their institute is that ‘Everybody deserves high quality healthcare, and that understanding the nature of people’s needs and preferences is integral to providing a high level of care’ [3]*.* Children and adolescents form a distinct patient group, whose needs and preferences have been relatively under-represented in published research [4, 5]. In recognition of this, the Picker Institute assisted in developing the first evidence-based patient-reported experience measure [6] and conducting the first children’s national UK survey in a decade [3, 7]. This showed that healthcare professionals (HCPs) do not always communicate well, leaving children and adolescents under-involved and unsure who to ask when concerned [7, 8]. Children’s and adolescents’ experiences of HCPs often fail to meet legal and professional standards set to protect children’s rights [9, 10]. The UK Royal College of Paediatrics and Child Health (RCPCH) described some of the survey results as ‘worrying’ [11] and made a strategic decision to involve children and adolescents in all matters that impact on their care [12].

Recent qualitative studies have shown how HCPs may influence children’s and adolescents’ experiences. Poor communication can lead to fear and frustration, missed appointments and poorer clinical outcomes [13–16]. The quotation at the head of this article exemplifies this. By the age of 12, Kelvin had undergone multiple urological procedures and became very familiar with indwelling catheters. The nurse, while attempting to empty his catheter bag, rejected Kelvin’s experience and needs by imposing her own experience onto him [1]. While HCPs and parents act as an essential source of support and advocacy, research has shown that their experiences are an inadequate proxy for children’s and adolescents’ experiences [5, 17], and that children and adolescents are best placed to describe their own experiences and needs [18, 19].

A qualitative methodology named phenomenology is well suited to helping researchers examine patients’ subjective experiences. Researchers using this methodology interpret verbal accounts of personal experience to examine how ‘phenomena’, such as being a sick child, present themselves to subjects’ minds and bodies [20]. There has been qualitative research into children’s and adolescents’ experiences of illness, but this has not yet focussed specifically on interactions between children and adolescents and HCPs. In order to equip HCPs to provide care which is better aligned with patients’ needs, this study aims to explore children’s and adolescents’ *lived* experiences of HCPs, as directly described by them.

## Methods/design

This study will use scoping review methodology, conducted from a phenomenological stance, to map existing published literature in a comprehensive and systematic way in order to identify key concepts and gaps in research on the topic. Scoping reviews are increasingly popular for addressing broad, exploratory topics such as this one. Benefits of a scoping review include the ability to examine the breadth and depth of existing evidence from a diverse range of sources, iteratively refine the research question and search strategy and include a consultation exercise with key stakeholders. The flexibility of scoping review methodology allows a complex topic, such as patients’ experiences, to be explored rigorously by ensuring that each step and decision is taken systematically and transparently, based on a clearly stated purpose and rationale [21–23]. As with previous scoping review protocols, PROSPERO is not applicable, and a PRISMA-P checklist has been included (see Additional File 1).

The authors of this protocol are well suited to conduct this review because they have expertise both of providing children’s healthcare (GD, AT, MK) and of using scoping review methodology (GD, MK, TD). This protocol follows the six-step methodological framework defined by Arksey and O’Malley [21] and further described by Levac et al. [22] and Colquhoun et al. [23]. The six steps of this review include (1) defining the research question; (2) identifying relevant articles; (3) study selection; (4) charting the data; and (5) collating, summarising and reporting the results; and (6) a consultation exercise.

### Step 1: Defining the research question

When embarking on this review, the authors acknowledged the sparsity of evidence known to them and proceeded initially by asking *What is known about children’s and adolescents’ experiences of healthcare?* A pilot review of existing literature provided important information. First, HCPs have the strongest influence on children’s and adolescents’ experience of healthcare [1, 24]. Second, a variety of participants report children’s and adolescents’ experiences, from a parent only [25, 26], to an adult reflecting back on their childhood experience [27], to children and adolescents themselves [14]. Third, some authors merge the perspectives of multiple stakeholders, so ‘they’ or ‘many’ could refer to a child, adolescent and parent [28, 29] or children, adolescents and adult patients [30, 31].

These findings clarified the authors’ commitment to understanding what matters to children and adolescents, as reported by them directly, rather than their proxies. This helped the authors redefine the question as *What is known about children’s and adolescents’ experiences of healthcare professionals, from their present perspective?* Revising the question led to a number of decisions. The review will only include an individual child’s or adolescent’s account of their experience, expressed in their own words, rather than an adult describing childhood experiences in retrospect. It will not include amalgamated accounts of both children’s and adults’ perspectives. Since the review is exploratory and iterative in nature, the authors acknowledge that greater familiarity with the literature may require further refinement of the research question or pose additional questions.

### Step 2: Identify relevant articles

Electronic searches will be completed on Ovid MEDLINE, Embase, Scopus, CINAHL and Web of Science, from inception. These databases have been selected for their combined ability to generate a repertoire of evidence, specific to the research topic, across a range of interdisciplinary fields.

Under the guidance of a subject librarian, appropriate keywords and subject headings identified through piloting searches will be used and expanded to maximise the number of relevant articles. Subject headings will include hospitals/, community health services/, qualitative research/ and emotions/, whereas relevant keywords will include child*, adolescen*, opinion* and experience*. Searches will be restricted to English language, as translated texts can have a distorted meaning from the original [32]. The final search will run on Ovid MEDLINE and be converted, to accommodate for differences in functionality across the other four databases. The reference lists of all relevant articles will be reviewed.

The scope of this study will be limited to published literature, as pilot searches have shown this will provide ample material for analysis and this will be the most efficient and effective way of identifying relevant articles that are accessible in full texts [33–35]. All citations will be imported into the Mendeley Reference Manager and duplicates removed.

### Step 3: Study selection

To capture children’s and adolescents’ *lived* experiences in all their richness, and in keeping with phenomenological principles, presence of children’s and adolescents’ first-person direct quotations will be an essential selection criterion. This criterion has practical advantages because quotations are usually referenced to specific participants and/or presented within quotation marks and/or italics.

Articles will be selected based on the following eligibility criteria, regardless of the country of origin or date of publication.

#### Eligibility criteria

##### Inclusion criteria

1. Children and/or adolescents speaking about one or more HCPs, on one or more instances, from any past experience, through first-person direct quotation(s), where there has been direct contact between the two parties and where the child or adolescent was the patient or person receiving healthcare.
2. A HCP is defined as a member of the healthcare team with professional qualifications and training, such as qualified doctors, nurses, therapists, psychologists and social workers, regardless of their grade.
3. Children and/or adolescents are defined as persons up to 18 years, regardless of their health status or illness type.

##### Exclusion criteria

1. Adults (over 18 years) included in the study, with or without children and adolescents, as patient participants.
2. Children and/or adolescents speaking about HCP(s) not from memory of an experience as a patient. For example, a third-party description (e.g. parents).
3. Non-English language publications

#### Types of studies

Types of articles likely to meet the eligibility criteria will be those presenting original qualitative or mixed-methods research findings (or syntheses), within full-text research reports. The analysis will take a phenomenological stance; however, included articles will not be restricted to phenomenological research or any other specific qualitative methodology. Interviews, focus groups, observation studies or interactive workshops, either in person or virtual, will potentially be included. Editorials, commentaries, organisational reports, opinion pieces and white papers will not be included due to the lack of children’s and adolescents’ quotations. Books, conference abstracts or unpublished theses will not be included due to difficulty identifying and obtaining them.

#### The selection process

Articles will be selected systematically using a three-stage process. Stage 1: The titles and abstracts of all articles identified by the search will be reviewed by the first author. To validate the selection process, a minimum of 10% of articles will be reviewed by another author. This process will continue to the point of ‘sufficiency’, when the two reviewers have reached a shared understanding of how to apply the inclusion criteria and resolved any disagreements about inclusion. Stage 2: The first author will review all articles selected by title and abstract in full text and apply the eligibility criteria. As in stage 1, another author will second-screen articles to sufficiency, defined as having resolved any discrepancies and reached full agreement about how to apply the inclusion criteria. Stage 3: The first author will refer any articles where she is uncertain about inclusion for a second author to review. A further check on the appropriateness of the included texts will take place at the data charting stage. The number of studies included and excluded at each stage, along with reasons for exclusion, will be presented in a PRISMA flow diagram.

Methodological quality will not be assessed, in keeping with scoping review methodology.

### Step 4: Charting the data

#### Basic numerical analysis

Data charted for basic numerical analysis will include the year of publication, country of origin, number and age range of participants, methodology and methods. Contextual information such as the setting, health condition(s) and type of encounter (acute or chronic) will be charted.

#### Qualitative analysis: quotations

Individual quotations within each article, along with the child’s or adolescent’s age and gender, will be identified and charted by the first author. A minimum of two authors will review all quotations to confirm that it is appropriate to include them. If all quotations from an article were excluded by this process, that article would be excluded and the PRISMA diagram updated.

Charting will be done electronically. The authors will meet after charting the data from 10 articles to review the extracted variables and modify as needed.

### Step 5: Collating, summarising and reporting the results

#### Basic numerical analysis

Numerical data will be analysed using Microsoft Excel. Graphical illustrations will show the trend in years of publication, number of articles per country, number of participants per study (and the total number), age range of participants per study, frequency of methods and methodologies used and range of clinical settings, health condition(s) and types of encounters.

#### Qualitative analysis: quotations

The authors will work together to analyse the quotations thematically, using procedures described by Braun and Clarke [36] and supported by qualitative data analysis software (NVivo). We will complete this process reflexively by constantly asking ourselves, and each other, how our preconceptions are influencing the analytical process. We will achieve the aim of the study by presenting these findings, supported by illustrative quotations.

### Step 6: Consultation exercise

The consultation exercise, which aims to validate and elaborate findings, will consist of up to two focus groups with up to 10 children and adolescents per group. Participants will be inpatients in the Royal Belfast Hospital for Sick Children (RBHSC), aged 8–16, with normal cognitive function, who are willing to participate. The focus group(s) will be conducted in the RBHSC and will abide by conditions set by a research committee and governance office. The authors will compile a set of key quotations, present these to focus group participants and ask participants to discuss these in relation to their own experiences. We have chosen to present key quotations to uphold the richness of the empirical data, which will help participants to better connect with the discussion. The age range selected is based on the ease at which children are likely to engage with the material presented (8 or above) and the inpatient demographics (16 or below).

The focus group will start with an ‘ice-breaker’, where the researchers, children and adolescents play a bespoke healthcare description game, taking turns at being the ‘describer’. The ‘describer’ aims to get the ‘guessers’ to say the healthcare-related word without saying it themselves. Discussions will be audio-recorded and transcribed verbatim. The authors will make notes and keep a written reflective log during the focus group(s) and afterwards. Focus group findings will be disseminated along with the thematic findings.

#### Study findings and dissemination

The resulting thematic framework will consist of major and minor themes, presented with detailed descriptions, exemplar quotations and focus group comments. The discussion of the article will report limitations, challenges, any variances from the published protocol, findings in relation to other published literature, recommendations for future research and implications for practice. The study findings will be submitted for publication, along with the PRISMA extension for scoping reviews, to an appropriate journal [37].

## Discussion

We have presented our protocol, which offers feasible means for exploring, describing and identifying gaps in what is known about children’s and adolescents’ *lived* experiences of HCPs. This research will be conducted within the qualitative paradigm, take a phenomenological stance, and adopt the increasingly used scoping review methodology. It will have the inherent strengths and limitations of taking an interpretive epistemological position [38]. This review is not limited by country, disease type, HCP type or child’s age nor does it specifically seek to include children with special communicative needs. Therefore, findings will be transferable to a wide range of contexts but limited in depth and applicability to specific diseases. We limit the study’s scope to published literature in English language and acknowledge that although less balanced, our findings will be significant and informative.

The tabulated quotations will be taken out of the contexts of the original articles, which could make the analysis difficult, but this will be addressed by maintaining a detailed data extraction chart and referring to the source articles. The role of the primary researcher, including their methodological approach and lens for interpreting their data, will not be addressed during the analysis of quotes as it is beyond the scope of this review.

Most scoping reviews tend to omit the consultation stage [39], likely due to difficulties in recruiting participants and processing additional data, albeit, we view it as a worthy means to validate and elaborate on findings. Age-appropriate techniques will encourage engagement in this setting [40]; however, participants’ comments may contradict the preliminary results, making it harder to analyse and present the findings.

Publishing protocols a priori is a rigorous way of embarking on a literature review because it requires transparency in detailing the study methods and invites critical comments from peer reviewers. The authors will conduct this study as described here but in an exploratory and iterative manner. The research team will scrutinise any proposed refinement of the methods in relation to the aim and purpose of the review before approving it.

This study is unique as it will present a synthesis of children’s and adolescents’ *lived* experiences, from direct quotations. Findings will advance knowledge and assist HCPs to tailor their interpersonal skills to meet children’s and adolescents’ needs so that better healthcare can be provided. It will have implications for key stakeholders concerned with children’s healthcare, specifically, clinical educators, policy makers and guideline developers. Insights gained will lead to suggestions for further research.

## Supplementary information

**Additional file 1.** PRISMA-P 2015 Checklist.

## Data Availability

Not applicable

## Abbreviations

HCP: Healthcare professional
QUB: Queen’s University Belfast
RBHSC: Royal Belfast Hospital for Sick Children
RCPCH: Royal College of Paediatrics and Child Health
